# The Arabidopsis R2R3 MYB Transcription Factor MYB15 Is a Key Regulator of Lignin Biosynthesis in Effector-Triggered Immunity

**DOI:** 10.3389/fpls.2020.583153

**Published:** 2020-09-17

**Authors:** Seu Ha Kim, Pui Ying Lam, Myoung-Hoon Lee, Hwi Seong Jeon, Yuki Tobimatsu, Ohkmae K. Park

**Affiliations:** ^1^ Department of Life Sciences, Korea University, Seoul, South Korea; ^2^ Research Institute for Sustainable Humanosphere, Kyoto University, Uji, Japan

**Keywords:** *Arabidopsis thaliana*, MYB15, transcription factor, lignin biosynthesis, effector-triggered immunity

## Abstract

Lignin, a major component of the secondary cell wall, is important for plant growth and development. Moreover, lignin plays a pivotal role in plant innate immunity. Lignin is readily deposited upon pathogen infection and functions as a physical barrier that limits the spread of pathogens. In this study, we show that an Arabidopsis MYB transcription factor MYB15 is required for the activation of lignin biosynthesis genes such as *PAL*, *C4H*, *4CL*, *HCT*, *C3′H*, *COMT*, and *CAD*, and consequently lignin formation during effector-triggered immune responses. Upon challenge with the avirulent bacterial pathogen *Pst* DC3000 (*AvrRpm1*), lignin deposition and disease resistance were reduced in *myb15* mutant plants. Furthermore, whereas invading pathogens, together with hypersensitive cell death, were restricted to the infection site in wild-type leaves, they spread beyond the infected area in *myb15* mutants. The exogenous supply of the lignin monomer coniferyl alcohol restored lignin production and rescued immune defects in *myb15* plants. These results demonstrate that regulation at the transcriptional level is key to pathogen-induced lignification and that MYB15 plays a central role in this process.

## Introduction

Lignin is an organic polymer that serves as a major structural molecule of the secondary cell wall in plant vascular systems (Vanholme et al., 2010). Lignin confers rigidity and strength to the cell wall, enabling plants to grow upright. Lignin composition and content vary among different species, tissues, and cells, influencing plant physiology and development (Wang et al., 2013). Given its importance in plant development and biotechnological applications, lignin metabolism has been extensively studied in model plants, woody trees, and grass biomass crops (Vanholme et al., 2010; Wang et al., 2013; Liu et al., 2018; Umezawa, 2018). Lignin polymers are composed primarily of monolignols, coniferyl, sinapyl, and *p*-coumaryl alcohols, which are oxidatively cross-linked to generate guaiacyl (G), syringyl (S), and *p*-hydroxyphenyl (H) units, respectively (Boerjan et al., 2003). Monolignols are generated by the phenylpropanoid pathway *via* a series of enzyme reactions. Whereas *p*-coumaryl alcohol is synthesized by a subset of enzymes, phenylalanine ammonia lyase (PAL), cinnamate 4-hydroxylase (C4H), 4-coumarate-CoA ligase (4CL), cinnamoyl-CoA reductase (CCR), and cinnamyl alcohol dehydrogenase (CAD), the synthesis of coniferyl alcohol requires additional enzymes, *p*-hydroxycinnamoyl-CoA:quinate/shikimate *p*-hydroxycinnamoyltransferase (HCT), *p*-coumaroyl shikimate 3-hydroxylase (C3′H), caffeoyl shikimate esterase (CSE), and caffeoyl-CoA *O*-methyltransferase (CCoAOMT), and the synthesis of sinapyl alcohol requires two more enzymes, ferulate-5-hydroxylase (F5H) and caffeic acid *O*-methyltransferase (COMT) (Bonawitz and Chapple, 2010; Miedes et al., 2014; Vanholme et al., 2019).

Plants are equipped with a multilayered immune system to counteract the invasion of pathogens (Jones and Dangl, 2006). Pattern-triggered immunity (PTI) and effector-triggered immunity (ETI) are the major defense mechanisms activated when plants sense attacking pathogens through the cell surface and cytosolic receptors, respectively. PTI is known as the basal defense and relies on the recognition of pathogen-associated molecular patterns (PAMPs) by pattern recognition receptors (Zhang and Zhou, 2010; Zipfel, 2014). Adapted pathogens have evolved effectors that are delivered into plant cells to overcome PTI, resulting in disease development (Alfano and Collmer, 2004; Boller and He, 2009; Dou and Zhou, 2012). As a countermeasure, plants in turn deploy resistance (R) proteins or intracellular receptors for the recognition of effectors, triggering a robust immune response, so-called ETI (Cui et al., 2015). ETI is frequently associated with programmed cell death (PCD) termed the hypersensitive response (HR) (Greenberg et al., 1994; Coll et al., 2010). PTI and ETI activate overlapping responses, including transcriptional reprogramming, ion fluxes, oxidative burst, protein kinase activation, and cell wall remodeling such as lignification (Nicholson and Hammerschmidt, 1992; Torres, 2010; Tsuda and Katagiri, 2010).

Lignification is induced in response to biotic stress and plays an important role in disease resistance (Nicholson and Hammerschmidt, 1992; Sattler and Funnell-Harris, 2013). A number of genetic studies on monolignol biosynthetic genes have been performed in diverse plants and have shown positive correlations between the expression of lignin biosynthetic genes and disease resistance (Sattler and Funnell-Harris, 2013; Miedes et al., 2014). In some opposite cases, disruption of monolignol biosynthesis increased resistance to pathogens, but this effect was mostly due to changes in the production of defense-related metabolites and hormones (Maury et al., 2010; Gallego-Giraldo et al., 2011). In a recent study, we demonstrated that pathogen-induced lignification leads to spatial restriction of pathogens and enhances disease resistance (Lee et al., 2019). This response was dependent on Casparian strip membrane domain protein (CASP)-like proteins (CASPLs), CASPL1D1 and CASPL4D1, suggesting that lignin and CASPLs together generate a Casparian strip-like diffusion barrier that prevents the spread of pathogens (Roppolo et al., 2011; Lee et al., 2019; Tobimatsu and Schuetz, 2019).

MYB proteins represent a large family of transcription factors that contain the conserved MYB DNA-binding domain (Dubos et al., 2010; Liu et al., 2015). MYB domain consists of up to four imperfect repeats (R) of 50 to 53 amino acid sequences. Depending on the number of repeats, MYB proteins have been grouped into four classes: 1R (R1/2, R3-MYB), 2R (R2R3-MYB), 3R (R1R2R3-MYB), and 4R (four R1/R2-like-MYB). Among these, R2R3-MYB proteins constitute the largest class of MYB transcription factors in plants and have been further divided into 28 subgroups (Stracke et al., 2001). A number of R2R3-MYB transcription factors have been associated with lignin biosynthesis in the secondary cell wall of vascular tissues (Zhao and Dixon, 2011; Liu et al., 2015). In Arabidopsis, R2R3-MYBs MYB46 and MYB83 activate the expression of *MYB58*, *MYB63*, and *MYB85*, which then specifically upregulate lignin biosynthesis genes, such as *PAL1*, *C4H*, *4CL1*, *HCT*, *C3′H*, and *CCoAOMT1* (Zhong et al., 2008; Zhou et al., 2009). In addition to extensive deposition in the secondary cell wall, lignin is an essential component of the Casparian strip, a diffusion barrier in the root endodermis (Roppolo et al., 2011; Naseer et al., 2012; Lee et al., 2013). For assembly of the Casparian strip, CASPs are initially expressed in the plasma membrane of root endodermal cells and this recruits other Casparian strip components such as peroxidase 64 (PER64) and enhanced suberin 1 (ESB1), which are required for precise lignin polymerization (Roppolo et al., 2011; Naseer et al., 2012; Hosmani et al., 2013; Lee et al., 2013). Genetic studies have led to the identification of MYB36, as a transcription factor that directly activates the expression of Casparian strip-associated genes *CASPs*, *PER64*, and *ESBs*, and is therefore required for Casparian strip formation (Kamiya et al., 2015; Liberman et al., 2015).

It has recently reported that the R2R3-MYB transcription factor MYB15 positively regulates the PAMP flg22-induced lignification and basal immunity (Chezem et al., 2017). Given that ETI is characterized by a substantial accumulation of lignin (Lee et al., 2019), this raises the question as to whether MYB15 also controls ETI-associated lignification. In this study, we show that MYB15 is indeed required for the expression of lignin biosynthesis genes and development of a lignin-based barrier during the ETI response. These results demonstrate that MYB15 is a major regulator of pathogen-induced lignification in plant innate immunity.

## Materials and Methods

### Plant Materials

*Arabidopsis thaliana* (ecotype Columbia, Col-0) plants were grown at 23°C and 70% relative humidity under long-day conditions (16 h light/8 h dark) with a light intensity of 75 μE/m^2^/s. The mutant lines used in this study are *myb15-1* (SALK_151976), *myb15-2* (SK2722), and *amiCASPL1D1 caspl4d1* (Lee et al., 2019). T-DNA insertion sites were confirmed by sequencing using gene-specific primers (**Supplementary Table S1**).

### Plant Treatments

Bacterial strains were grown on King’s B agar medium containing 50 μg/ml kanamycin and 100 μg/ml rifampicin at 28°C (King et al., 1954). Plants were grown at 23°C and 70% relative humidity under short-day conditions (8 h light/16h dark) with a light intensity of 75 μE/m^2^/s for 4 weeks. Pathogen and chemical treatments were performed as previously described (Lee et al., 2019). For treatment with *Pseudomonas syringae*, plant leaves were syringe-infiltrated with 10 μl of 10 mM MgCl_2_ (mock) or a bacterial cell suspension at 10^6^ cfu/ml for bacterial growth analysis and at 10^8^ cfu/ml for all other experiments. For coniferyl alcohol treatment, coniferyl alcohol was dissolved in 100% dimethyl sulfoxide (DMSO; Duchefa) to prepare a 50 mM stock solution, which was diluted with distilled water to a final concentration of 50 μM, and syringe-infiltrated into leaves. Chemical-treated leaves were air-dried for 2 h before pathogen inoculation.

### Bacterial Growth Assay

Bacterial growth was determined as previously described (Lee et al., 2019). Four-week-old plants were syringe-inoculated with *P. syringae* pv. *tomato* (*Pst*) DC 3000 (*AvrRpm1*) at 10^6^ cfu/ml (OD_600_ = 0.001). Two leaf discs (5 mm in diameter) per leaf were taken and pooled as a single replicate. The pooled leaf discs were ground in 10 mM MgCl_2_, and bacterial growth was determined by serial dilution plating on King’s B agar medium containing 50 μg/ml kanamycin and 100 μg/ml rifampicin. Experiments were repeated three times with biologically independent samples.

### Gene Expression Analysis

Total RNAs were extracted using TRIzol reagent and reverse-transcribed into cDNAs using PrimeScript™ RT Reagent Kit (TaKaRa). Quantitative real-time PCR was performed using KAPA SYBR FAST qPCR Master Mix (Kapa Biosystems) with gene-specific primers (**Supplementary Table S1**) in a LightCycler 480 system (Roche). The expression levels of genes were normalized to a reference gene *Actin2* and analyzed using LC480Conversion and LinRegPCR software (Heart Failure Research Center). Experiments were repeated at least three times with biologically independent samples.

### Phloroglucinol Staining

Phloroglucinol staining was performed at room temperature as previously described (Lee et al., 2019). Leaves were dehydrated in 100% ethanol overnight and rehydrated in a graded series of ethanol (75, 50, and 25%) and water for 1 h each. Rehydrated leaves were then stained with 3% phloroglucinol (Sigma-Aldrich) dissolved in 30% HCl for 1 min. The stained leaves were observed under an optical microscope (Leica EZ4E). Experiments were repeated three times with similar results.

### Acetyl Bromide-Based Lignin Quantification

Lignin content was determined by the acetyl bromide-based method as previously described (Lee et al., 2019). Leaves were ground to a fine powder in liquid nitrogen, and the dried samples (1–3 mg) were washed serially with 70% ethanol, chloroform/methanol (1:1 v/v), and acetone. The washed pellets were completely dried at 55°C and treated with 1 ml of 25% acetyl bromide in acetic acid at 70°C for 1 h with vortexing every 10-min intervals. Samples were cooled in ice and centrifuged for 1 min. The supernatants (100 μl) were transferred to new tubes and mixed with 2 M NaOH (400 μl), 0.5 M hydroxylamine hydrochloride (70 μl), and acetic acid (430 μl). The prepared solutions were then transferred to 96-well microplates, and the absorbance was measured using Microplate Reader (Molecular Devices) at 280 nm. The content of acetyl bromide soluble lignin (% ABSL) was calculated using Beer’s Law (Kapp et al., 2015). The extinction coefficient used for Arabidopsis was 15.69 l/g cm (Foster et al., 2010). Experiments were repeated three times with similar results.

### Thioacidolysis-Based Lignin Composition Analysis

Ground leaf tissues were washed serially with water, 80% ethanol, and acetone, and freeze dried. The washed leaf tissues (~3 mg) were then subjected to analytical thioacidolysis according to the method described by Yamamura et al. (2012). The released lignin monomers were derivatized with *N*,*O*-bis(trimethylsilyl)acetamide and quantified by gas chromatography-mass spectrometry, using 4,4′-ethylenebisphenol as an internal standard (Yue et al., 2012). Experiments were repeated two times with similar results.

### Microscopic Analysis of Bacterial Pathogens

Leaves were infiltrated with GFP-*Pst* DC3000 (*AvrRpt2*) at 10^8^ cfu/ml and incubated for 2 days. Images for bacterial colonization were taken using a confocal microscope (Zeiss LSM 700). Bacterial spreading was assessed by 15 to 25 randomly selected fields of view from at least three leaves per genotype/treatment.

### Statistical Analysis

Statistical analyses were performed using GraphPad Prism (v. 8.0). Significant differences between values were analyzed by one-way ANOVA with Tukey’s HSD test or unpaired Student’s *t*-test for multiple comparisons or single comparisons, respectively.

## Results

### MYB15 is Required for the Expression of Lignin Biosynthesis Genes in ETI

**Figure 1:**
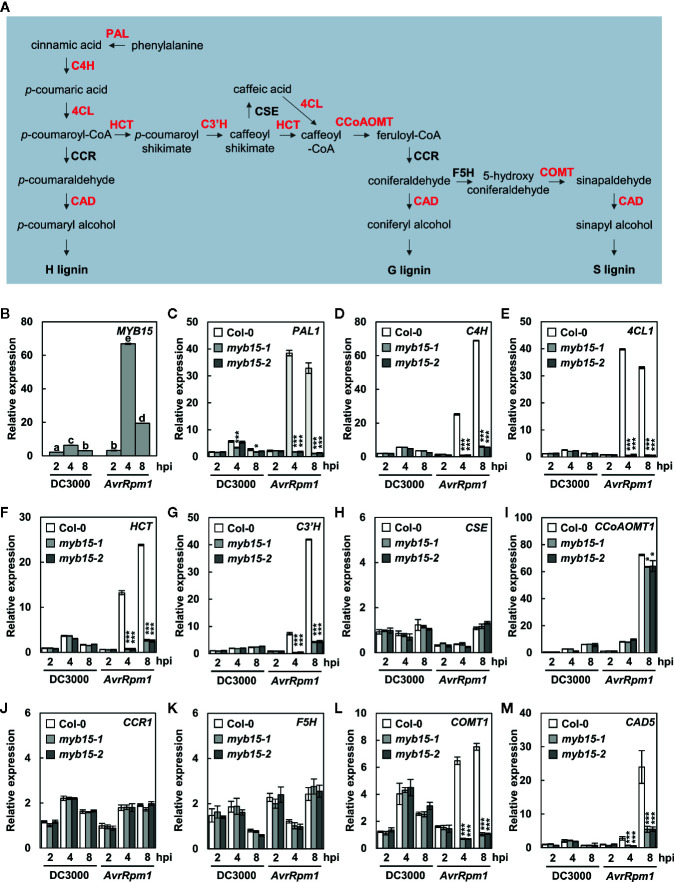
MYB15 positively regulates the expression of lignin biosynthetic genes in ETI. **(A)** The lignin biosynthetic pathway. The enzymes in red color show MYB15-dependent gene expression. **(B)** Relative expression of *MYB15* as compared to mock (10 mM MgCl_2_) treatment after infection with *Pst* DC3000 and *Pst* DC3000 (*AvrRpm1*). Data are means ± SD (*n* = 4). Significant differences are indicated by different letters (Tukey’s HSD test; *P* < 0.05). **(C–M)** Relative expression of lignin biosynthesis genes, *PAL1* **(C)**, *C4H* **(D)**, *4CL1* **(E)**, *HCT* **(F)**, *C3′H* **(G)**, *CSE* **(H)**, *CCoAoMT1* **(I)**, *CCR1* **(J)**, *F5H* **(K)**, *COMT1* **(L)**, and *CAD5* **(M)**, as compared to mock (10 mM MgCl_2_) treatment after infection with *Pst* DC3000 and *Pst* DC3000 (*AvrRpm1*). Data are means ± SD (*n* = 4). Asterisks indicate significant differences from the respective Col-0 (*t* test; *P < 0.05; ***P < 0.001). Four-week-old leaves were syringe-infiltrated with bacteria at 10^8^ cfu/ml. hpi, hours post-inoculation; DC3000, *Pst* DC3000; *AvrRpm1*, *Pst* DC3000 (*AvrRpm1*).

Angiosperm lignin is typically composed of G and S lignin units with very little H units, and the role of G lignin in disease resistance has been demonstrated in Arabidopsis (Miedes et al., 2014; Chezem et al., 2017). Chezem et al. (2017) showed that MYB15 activates genes involved in lignin biosynthesis in basal immunity. Here we examined whether MYB15 also regulates lignin biosynthesis during incompatible plant-pathogen interactions. T-DNA insertion mutants *myb15-1* and *myb15-2* were obtained (Chezem et al., 2017) and checked for the expression of monolignol biosynthesis genes in response to virulent *Pst* DC3000 and avirulent *Pst* DC3000 (*AvrRpm1*) treatments (**Figure 1A**). In the case of *CCR*, expression of two genes, *CCR1* and *CCR2*, was analyzed, as they exhibited differential expression patterns in response to bacterial infection (Lauvergeat et al., 2001). *MYB15* itself and most of monolignol biosynthesis genes, including *PAL1*, *C4H*, *4CL1*, *HCT*, *C3′H*, *CCoAoMT1*, *COMT1*, and *CAD5*, were highly expressed in wild-type Col-0 plants by *Pst* DC3000 (*AvrRpm1*) treatment, compared to *Pst* DC3000 treatment (**Figures 1B–M**, **Supplementary Figure S1**). However, the activation of these genes was almost abolished in *myb15* mutants, except *CCoAoMT1* showing a small, albeit significant, decrease. In contrast, *CSE*, *CCR1*, *CCR2*, and *F5H* showed little response to *Pst* DC3000 (*AvrRpm1*) treatment in both wild-type and *myb15* plants. These results suggest that MYB15 is a key regulator for the expression of most lignin biosynthetic genes during ETI.

Next, we examined the expression of *CASPL1D1* and *CASPL4D1* in wild-type and *myb15* plants. They were significantly upregulated in both *Pst* DC3000 (*AvrRpm1*)-treated wild-type and *myb15* plants, with slight reduction (~15%) of *CASPL1D1* expression in *myb15* mutants (**Supplementary Figure S1**). This implies that *CASPL1D1* and *CASPL4D1* are unlikely among the MYB15 target genes.

### Pathogen-Induced Lignification is Dependent on MYB15

**Figure 2:**
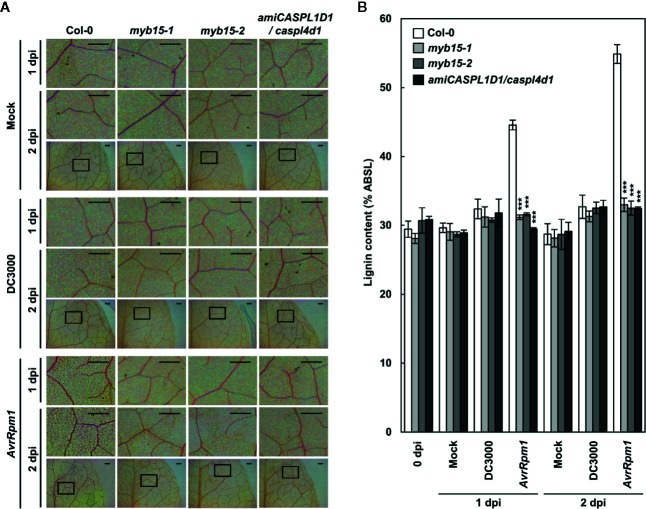
MYB15 is required for pathogen-induced lignification. **(A)** Phloroglucinol staining of wild-type, *myb15*, and *amiCASPL1D1 caspl4d1* leaves infected with *Pst* DC3000 and *Pst* DC3000 (*AvrRpm1*). The upper images are enlarged ones of the lower boxes at 2 dpi. Scale bars, 100 μm. **(B)** Quantification of lignin content in wild-type, *myb15*, and *amiCASPL1D1 caspl4d1* leaves infected with *Pst* DC3000 and *Pst* DC3000 (*AvrRpm1*). Data are means ± SD (*n* = 4; 3-9 leaves each). Asterisks indicate significant differences from the respective Col-0 (*t* test; ***P < 0.001). Four-week-old leaves were syringe-infiltrated with bacteria at 10^8^ cfu/ml. dpi, days post-inoculation; DC3000, *Pst* DC3000; *AvrRpm1*, *Pst* DC3000 (*AvrRpm1*).

Lignin accumulation in pathogen-inoculated plants was visualized by phloroglucinol staining and quantified by the acetyl bromide assay. We previously showed that pathogen-induced lignification requires CASPLs, CASPL1D1 and CASPL4D1, and is compromised in *amiCASPL1D1 caspl4d1* double mutant plants, which were prepared by crossing the *caspl4d1-1* knockout mutant (SALK_201606) and an artificial miRNA-overexpressing *amiCASPL1D1* knockdown line (Lee et al., 2019). Using the *amiCASPL1D1 caspl4d1* mutant as a negative control, we observed the characteristic purple-red staining in the extracellular space of mesophyll cells in wild-type, but not in *myb15* leaves, upon *Pst* DC3000 (*AvrRpm1*) infection (**Figure 2A**). Consistent with the staining data, *Pst* DC3000 (*AvrRpm1*) treatment led to a large increase in lignin content only in wild-type plants (**Figure 2B**).

**Figure 3:**
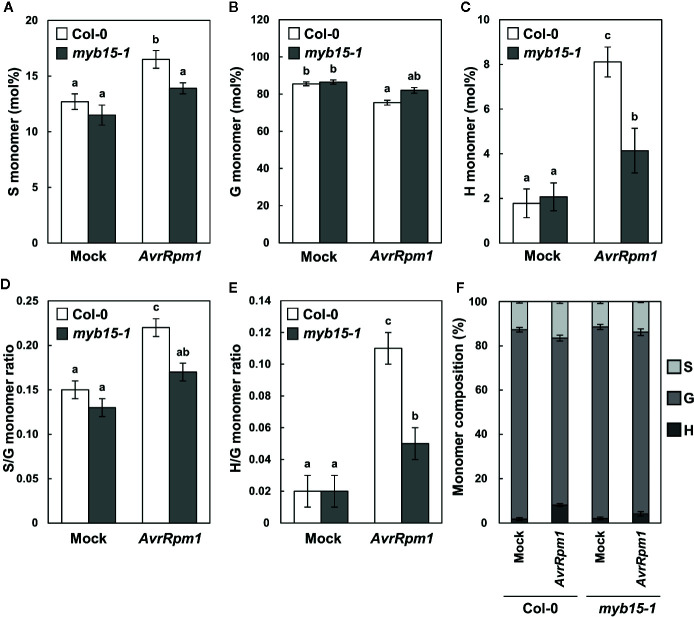
Thioacidolysis analysis of monolignol composition. **(A–C)** Relative abundance (mol%) of syringyl, S **(A)**, guiacyl, G **(B)**, and *p*-hydroxyohenyl, H **(C)** monomers released from wild-type and *myb15* cell walls upon thioacidolysis degradation. **(D, E)** Ratios of S to G **(D)** and H to G **(E)** monomers. **(F)** Composition of S, G, and H monomers. Graphs were constructed using data presented in **Table 1**. Data are means ± SD of triplicates of 240–260 pooled leaves. Significant differences are indicated by different letters (Tukey’s HSD test; *P* < 0.05). Four-week-old leaves were treated with mock (10 mM MgCl_2_) or 10^8^ cfu/ml *Pst* DC3000 (*AvrRpm1*) for 2 days.

Lignin composition in *myb15* mutant cell walls compared to wild-type was evaluated by analytical thioacidolysis, which releases β–*O*–4 linked lignin monomers (Yue et al., 2012). Interestingly, the proportion of H units largely increased over 4-fold and S units also showed the increase, albeit to a lesser extent, in *Pst* DC3000 (*AvrRpm1*)-treated wild-type leaves, compared to mock-treated wild-type (**Table 1**, **Figure 3**). The abundance of H and S monomers was considerably reduced in pathogen-infected *myb15* mutant, further supporting MYB15-dependent lignin biosynthesis. In contrast, the proportion of the most abundant G monomers was not significantly altered in both wild-type and *myb15* leaves. Accordingly, S/G and H/G ratios were significantly increased by 1.5-fold (0.15 to 0.22) and 5.5-fold (0.02 to 0.11), respectively, in pathogen-infected wild-type leaves.

**Table 1:** Thioacidolysis analysis of monolignol composition in wild-type and *myb15* leaves after mock or *Pst* DC3000 (*AvrRpm1*) treatments.

	Col-0+mock	Col-0+*AvrRpm1*	*myb15-1+*mock	*myb15-1*+A*vrRpm1*
**S (mol%)**	12.7 ± 0.7	16.5 ± 0.8	11.5 ± 0.9	13.9 ± 0.5
**G (mol%)**	85.5 ± 1.0	75.4 ± 1.3	86.5 ± 1.1	82.0 ± 1.5
**H (mol%)**	1.78 ± 0.64	8.11 ± 0.67	2.07 ± 0.62	4.14 ± 1.00
**S/G**	0.15 ± 0.01	0.22 ± 0.01	0.13 ± 0.01	0.17 ± 0.01
**H/G**	0.02 ± 0.01	0.11 ± 0.01	0.02 ± 0.01	0.05 ± 0.01

Data are means ± SD of triplicates of 240–260 pooled leaves. Four-week-old leaves were treated with mock (10 mM MgCl_2_) or 10^8^ cfu/ml Pst DC3000 (AvrRpm1) for 2 days.

### MYB15-Mediated Lignification is Required for ETI Responses

**Figure 4:**
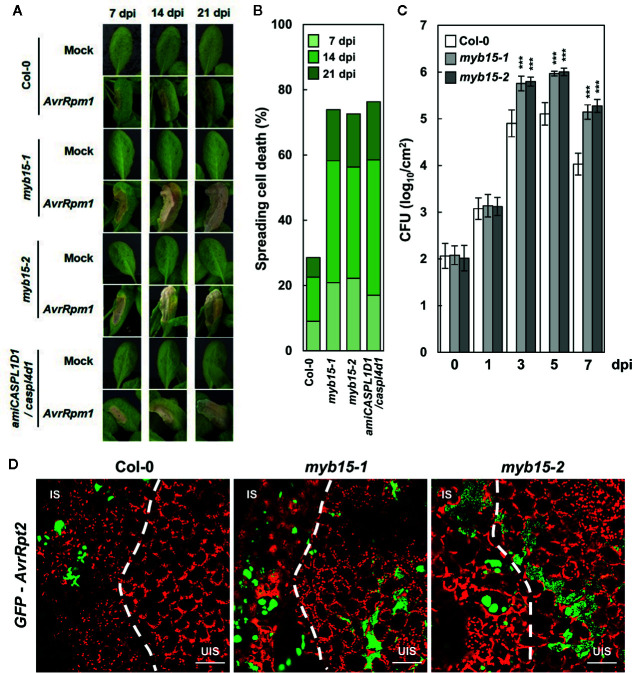
MYB15 is important for immune responses against avirulent bacterial pathogens. **(A)** Cell death phenotypes of wild-type, *myb15*, and *amiCASPL1D1 caspl4d1* leaves infected with *Pst* DC3000 (*AvrRpm1*). **(B)** Quantification of leaves (*n* ≥ 30) with spreading cell death as in **(A)**. **(C)** Measurements of *Pst* DC3000 (*AvrRpm1*) growth. Data are means ± SD (*n* = 3). Asterisks indicate significant differences from the respective Col-0 (*t* test; ***P < 0.001). **(D)** Colonization patterns of GFP-*Pst* DC3000 (*AvrRpt2*) in wild-type and *myb15* mutants at 2 dpi. Four-week-old leaves were syringe-infiltrated with bacteria at 10^6^ cfu/ml for growth assay and at 10^8^ cfu/ml for other experiments. Experiments were repeated three times with similar results. dpi, days post-inoculation; *AvrRpm1*, *Pst* DC3000 (*AvrRpm1*); IS, infected site; UIS, uninfected site. White dashed lines indicate the boundary between IS and UIS. Scale bars, 100 μm.

In our previous study, pathogen-induced lignification was vital for restricting HR PCD and enhancing disease resistance (Lee et al., 2019). In this context, it was determined whether MYB15 regulates ETI responses to *Pst* DC3000 (*AvrRpm1*) challenge. Whereas HR PCD was restricted in wild-type leaves, cell death gradually spread over the infection site in *myb15* leaves, similar to that observed in *amiCASPL1D1 caspl4d1* plants (**Figures 4A, B**). In addition, bacterial growth was increased, and therefore, disease resistance was decreased in *myb15* plants, compared to wild-type (**Figure 4C**). Consistent with the role as a physical barrier, we previously demonstrated that lignin prevents the spread of invading pathogens (Lee et al., 2019). Thus, we infiltrated wild-type and *myb15* leaves with green fluorescent protein (GFP)-labeled *Pst* DC3000 (*AvrRpt2*) and examined bacterial colonization. Whereas fluorescent bacteria were restricted to the infection site in wild-type, they spread and were detected in the nearby uninfected region in *myb15* mutants in most microscope fields of view (**Figure 4D**). These results demonstrate that MYB15 regulates ETI-associated immune responses.

**Figure 5:**
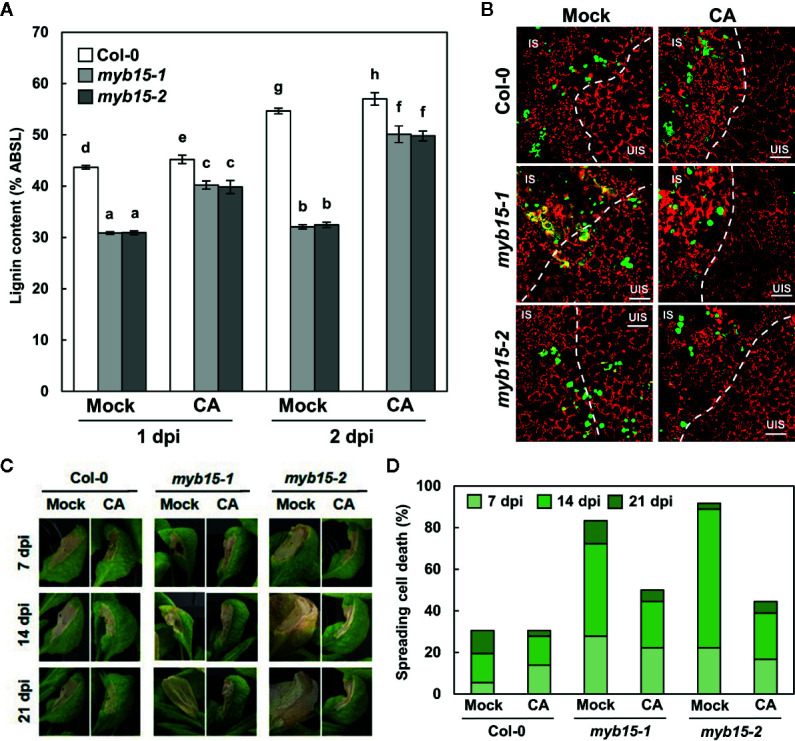
MYB15-mediated lignification restricts pathogens and HR PCD. **(A)** Quantification lignin content after coniferyl alcohol pretreatment and *Pst* DC3000 (*AvrRpm1*) infection. Data are means ± SD (*n* = 4; 3–9 leaves each). Significant differences are indicated by different letters (Tukey’s HSD test; *P* < 0.05). **(B)** Colonization patterns of GFP-*Pst* DC3000 (*AvrRpt2*) in coniferyl alcohol-pretreated wild-type and *myb15* mutants at 2 dpi. IS, infected site; UIS, uninfected site. The white dashed lines indicate the boundary between IS and UIS. Scale bars, 100 μm. **(C)** Cell death phenotypes of leaves after coniferyl alcohol pretreatment and *Pst* DC3000 (*AvrRpm1*) infection. **(D)** Quantification of leaves (*n* ≥ 30) with spreading cell death as in **(C)**. Four-week-old leaves were syringe-infiltrated with bacteria at 10^8^ cfu/ml. Experiments were repeated three times with similar results. dpi, days post-inoculation; CA, coniferyl alcohol.

Since MYB15 leads to the production of several phenolics, including lignin (Chezem et al., 2017), it was assessed whether defective phenotypes of *myb15* mutants are due to lignin deficiency. For this, plants were supplied with coniferyl alcohol, the monolignol composing G lignin units, prior to bacterial infiltration. The exogenous application of coniferyl alcohol restored lignin production in *myb15* mutants (**Figure 5A**). Moreover, coniferyl alcohol treatment rescued immune defects in *myb15* mutants, preventing the spread of HR PCD and invading pathogens (**Figures 5B–D**). These results demonstrate that MYB15 induces lignification, and *via* this process, controls ETI responses.

## Discussion

Previously, we showed that intense lignin deposition is induced at the infection site of leaves challenged with avirulent bacterial pathogens (Lee et al., 2019). It led to spatial restriction of invading pathogens, supporting the role of lignin as a mechanical barrier. The accumulation of lignin was dependent on CASPLs, CASPL1D1 and CASPL4D1. suggesting that lignin is associated with the Casparian strip-like structure, as lignin and CASPs were known to be the main components of the Casparian strip, a diffusion barrier in the root endodermis (Roppolo et al., 2011; Naseer et al., 2012; Lee et al., 2013). In fact, the endodermal Casparian strip in roots was shown to play a role in defense against root‐parasitic nematode species, the cyst nematode *Heterodera schachtii* and the root‐knot nematode *Meloidogyne incognita* (Holbein et al., 2019).These results suggest that the Casparian strip may also function in defense against soil-borne pathogens.

Lignin formation is a rapid immune response, in which regulation of involving gene expression could be critical and occur at several levels i.e., transcriptional, post-transcriptional, translational, and post-translational levels. Chezem et al. (2017) demonstrated the transcriptional regulation of lignin biosynthesis genes and identified MYB15 as a regulator for their activation in basal immunity. MYB15 activated genes involved in G lignin biosynthesis through binding to AC element- and MYB-responsive element (SMRE)-containing promoter regions. Accordingly, we sought to determine whether ETI-associated lignification is also controlled at the transcriptional level, and if so, whether MYB15 is the transcription factor for the regulation of lignin biosynthesis genes in ETI, as in PTI. We found that *MYB15* is readily activated and required for the expression of a number of lignin biosynthetic genes, *PAL1*, *C4H*, *4CL1*, *HCT*, *C3′H*, *CCR2*, *COMT1*, and *CAD5*, in response to *Pst* DC3000 (*AvrRpm1*) treatment, which is similar to prior finding (Chezem et al., 2017).

CCR is the first enzyme specific to the monolignol pathway, playing a critical role in lignin biosynthesis. Among multiple *CCR* genes described in Arabidopsis, *CCR1* was highly expressed in all tissues examined, whereas *CCR2* transcripts were barely detected in tissues but accumulated rapidly during the incompatible interaction with *Xanthomonas campestris* pv. *campestris*, suggesting that CCR1 and CCR2 may be differentially involved in development and pathogen responses (Lauvergeat et al., 2001; Raes et al., 2003). In contrast to the earlier study, both *CCR1* and *CCR2* were not much responsive to pathogens. Other genes, including *PAL1*, *C4H*, *4CL1*, *HCT*, *C3′H*, *COMT1*, and *CAD5*, were strongly activated upon *Pst* DC3000 (A*vrRpm1*) infection and MYB15-dependent. However, *CCoAOMT1* was highly expressed by *Pst* DC3000 (*AvrRpm1*) treatment but showed a weak dependency on MYB15, suggesting that other transcription factors may function in concert with MYB15 for *CCoAOMT1* activation. Furthermore, *CSE* and *F5H* directing the metabolic flow toward G and S lignin production, respectively (Meyer et al., 1998; Vanholme et al., 2013), showed very little increase in expression in response to *Pst* DC3000 (*AvrRpm1*) treatment. This is different from the earlier study, in which both *CSE* and *F5H* were upregulated in response to flg22 (Chezem et al., 2017). We cannot rule out the possibility that any discrepancy between this and previous studies is due to differences in elicited immune responses (flg22 vs. *Pst* DC3000 (*AvrRpm1*)) and developmental stages (seedlings vs. adult plants). Further study will be necessary to investigate whether those unresponsive genes may be regulated by other mechanisms e.g., at the post-transcriptional, translational, and/or post-translational levels. In fact, there have been reports that miRNAs and phosphorylation regulate the expression and activity of enzymes involved in lignin formation (Wang et al., 2015; Sun et al., 2018).

In line with gene expression results, lignin content was largely increased by *Pst* DC3000 (*AvrRpm1*) treatment in a MYB15-dependent manner. Although the G lignin ratio remained little changed before and after pathogen treatment, the portion of G units in the increased lignin was still predominant (~80%) and this increase was not seen in *myb15* leaves. In the previous study (Chezem et al., 2017), the G lignin ratio even decreased after flg22 treatment. However, in the same context, the G lignin content increased in wild-type after flg22 treatment and compared to *myb15*. Previous and our data suggest that G lignin is important for MYB15-mediated disease resistance. On the other hand, H units were present in a tiny fraction in the wild-type control as is already known, but the H/G ratio was significantly increased in *Pst* DC3000 (*AvrRpm1*)-infected wild-type but much less in *myb15* leaves. This is in contrast to the G lignin ratio, which showed little fluctuation. The S/G ratio also showed a slight increase in the infected wild-type, and at least part of this increase required MYB15. The lignin barrier constructed in response to pathogen infection may be relatively enriched in H units and probably, to a lesser extent, in S units, compared to normal lignins produced in leaf cell walls. Many reports have revealed that lignin synthesized in response to stresses, the so-called “stress lignin”, has the characteristic of higher amounts of H units (Lange et al., 1995; Cesarino, 2019). H unit enrichment of stress lignin fits with the notion that H lignin requires fewer catalytic steps in its biosynthesis than other lignin units, and therefore, can trigger a rapid response. Our finding would be an important addition to those of previous studies supporting that the H unit is an essential constituent of stress lignin. Further investigation is needed to determine how a small fraction of H units in the lignin barrier contributes to disease resistance in terms of structure and function. Taken together, these results demonstrate that MYB15 plays a central role in lignification during immune responses.

Arabidopsis MYB58 and MYB63 are the regulators of lignin biosynthesis during secondary cell wall formation and their overexpression led to activation of genes in the lignin biosynthetic pathway, particularly substantial induction of G lignin biosynthesis genes, *PAL1*, *C4H*, *4CL1*, *C3′H*, *HCT*, and *CCoAOMT1* (Zhou et al., 2009). MYB15 and MYB58/63 belong to subgroups 2 and 3 of R2R3 MYBs, respectively, and they are closely related, according to the phylogeny of MYB transcription factors (Stracke et al., 2001; Liu et al., 2015), implying that MYB15 in defense is the counterpart of MYB58 and MYB63 in development for lignin biosynthesis. Unlike lignin biosynthesis genes, *CASPL1D1* and *CASPL4D1* expression showed little dependency on MYB15, suggesting the involvement of additional transcription factors in pathogen-induced lignification. In the root endodermis, MYB36 was identified as the transcription factor activating Casparian strip-associated genes *CASPs*, *PER64*, and *ESBs* (Kamiya et al., 2015; Liberman et al., 2015). Microarray analysis of genome-wide gene expression in roots of *myb36* mutants revealed that 23 genes are positively regulated by MYB36, including Casparian strip-associated genes but not lignin biosynthesis genes. It would be worthwhile examining whether MYB15 is involved in lignin biosynthesis in the endodermal Casparian strip, and conversely, whether MYB36 is involved in the regulation of lignin structure-forming genes during immune responses.

## Data Availability Statement

The raw data supporting the conclusions of this article will be made available by the authors, without undue reservation.

## Author Contributions

OP conceived the project. SK and OP designed the experiments, analyzed the data, and wrote the manuscript. SK performed most of the experiments. PL and YT performed thioacidolysis analysis. M-HL and HJ participated in lignin staining and quantification experiments. All authors contributed to the article and approved the submitted version.

## Funding

This work was supported by a Korea University grant, a Next-Generation BioGreen 21 Program (SSAC, PJ013202) from the Rural Development Administration, National Research Foundation of Korea (NRF) grants (2019R1A2C2003810; 2018R1A5A1023599, SRC) from the Korean government (MSIP), and research grants from the Japan Society for the Promotion of Science (JSPS KAKENHI, JP#16H06198 and JP#20H03044) and the Center for Exploratory Research on Humanosphere, RISH, Kyoto University.

## Conflict of Interest

The authors declare that the research was conducted in the absence of any commercial or financial relationships that could be construed as a potential conflict of interest.
