# Supplementary material for: The Arabidopsis R2R3 MYB Transcription Factor MYB15 Is a Key Regulator of Lignin Biosynthesis in Effector-Triggered Immunity
Source: Front Plant Sci. 2020 Sep 17;11:583153. doi: 10.3389/fpls.2020.583153 (PMC7527528; doi:10.3389/fpls.2020.583153)
Supplement: Supplementary file 1 [file DataSheet_1.pdf]

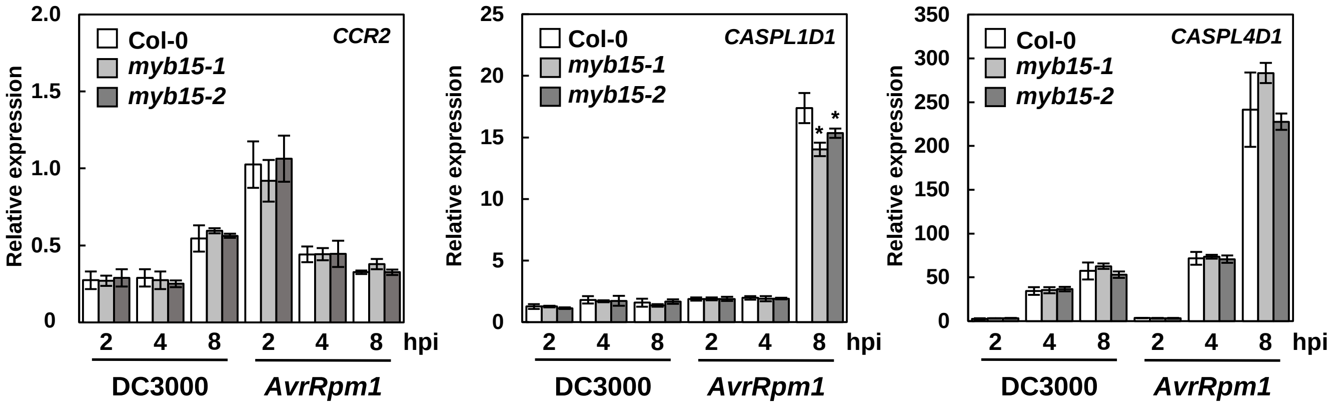

Figure S1. Relative expression of *CCR2*, *CASPL1D1*, and *CASPL4D1* as compared to mock (10 mM $MgCl_2$) treatment after infection with *Pst* DC3000 and *Pst* DC3000 (*AvrRpm1*). Data are means ± SD (n = 4). Asterisks indicate significant differences from the respective Col-0 (t test; $^*P < 0.05$). Four-week-old leaves were syringe-infiltrated with bacteria at 10^8 cfu/ml. hpi, hours post-inoculation; DC3000, *Pst* DC3000; *AvrRpm1*, *Pst* DC3000 (*AvrRpm1*).
