# Supplementary material for: The Arabidopsis R2R3 MYB Transcription Factor MYB15 Is a Key Regulator of Lignin Biosynthesis in Effector-Triggered Immunity
Source: Front Plant Sci. 2020 Sep 17;11:583153. doi: 10.3389/fpls.2020.583153 (PMC7527528; doi:10.3389/fpls.2020.583153)
Supplement: Supplementary file 2 [file DataSheet_2.pdf]

Table S1. Primers used in this study.

Gene/Line	Forward primer	Reverse primer
ACTIN2	CCTCAGCACATTCCAGCAGATGT	TGGGACTAAAACGCAAAACGAAAG
MYB15	CACCAAATACTTGGCAATAGATGG	GGCTTATGAGTGTCATCGAAGAAA
PAL1	GCTTAAGGCAGTGCTACCGA	ACCTTCACAAATCGCCGTGA
C4H	GGAAACGTTTGCAATTGATGATGT	GCAATTCCCCACTCGATAGACCAC
4CL1	CTAATGCCAAACTCGGTCAGGGATAC	CTCTTGTAAAACACAACCTGTTTCGAC
HCT	CTTCTCGTTTTGCAGGTGACTTTC	GTATTCTCAGGTCCTGATTTAG
C3'H	CATTGGTCTTCTATGGGATATG	CTTCGGTGAGGTAGCATTAGA
CSE	CCGATGGTATCCGCTGCTACATGGGTG	TTGTTCCCACTCTAGGCTTCCCTGTATATC TTTG
CCoAOMT1	GACCAATTGGTGAACGACAAA	CAACATCGACGAAACCACAAC
CCR1	GACCAAGTGCAAGGACGAGAA	ACGAGACATATTGAAACAGAGG
CCR2	AACTTCGTCCATTGAGGATTGGGAGGGAC	CCCACGCAGTATAGACCATCTATAGCCGT TG
F5H	CGTGACAATATCAAAGCAATCATCATGGACG	TCGAAATTGCTCCCTTTGAAATCCGGTAC
COMT1	GGTGATGCCATATTCATGAAGTG	CCGTGGAAGGAGATATGAGATAA
CAD5	TAAAGCCACCGTCGTTCACCAAAAGTTTG	AAGCTCCCCGTTATCACTTTCCTCCCAAG
CASPL1D1	CCAACTCACCGGCTCTTATAT	AGATATGACAGATTTTGCCCC
CASPL4D1	CGATGTCTATGCTTATAGATACATGC	AGAGAGACGAATGCGAAGAGA
SALK_151976	ACCAAAGAAGAGGAAGATGCTATC	TAAGAGATCTTGTTCCCCGC
SK2722	CCTCAACCATGGACATAGTAAC	TCGAGCTCGTTATTACGGATTC
